# Characterization and Biological Activity of Rutin Extracted from *Filipendula ulmaria* (L.) Maxim

**DOI:** 10.3390/biotech15010025

**Published:** 2026-03-23

**Authors:** Anna Vesnina, Violeta Le, Svetlana Ivanova, Anna Frolova, Irina Milentyeva, Victor Atuchin, Alexander Prosekov

**Affiliations:** 1 Natural Nutraceutical Biotesting Laboratory, Kemerovo State University, Kemerovo 650000, Russia; koledockop1@mail.ru (A.V.); ya808@yandex.ru (V.L.); frolova.anna.s@mail.ru (A.F.); irazumnikova@mail.ru (I.M.); 2 DNA Sequencing and Genomics Laboratory, Kemerovo State University, Krasnaya Street, 6, Kemerovo 650000, Russia; 3 Institute of NBICS-Technologies, Kemerovo State University, Krasnaya Street, 6, Kemerovo 650000, Russia; pavvm2000@mail.ru; 4 Department of TNSMD Theory and Methods, Kemerovo State University, Krasnaya Street, 6, Kemerovo 650000, Russia; 5 Research and Development Department, Kemerovo State University, Krasnaya Street, 6, Kemerovo 650000, Russia; 6 Department of Industrial Machinery Design, Novosibirsk State Technical University, Novosibirsk 630073, Russia; 7 Laboratory of Optical Materials and Structures, Institute of Semiconductor Physics, SB RAS, Novosibirsk 630090, Russia; 8 Laboratory of Biocatalysis, Kemerovo State University, Kemerovo 650043, Russia; a.prosekov@inbox.ru

**Keywords:** rutin, extraction, *Filipendula ulmaria* (L.) Maxim, bioactivity, biologically active substances, metabolic syndrome

## Abstract

In this work, *Filipendula ulmaria* (L.) Maxim, a perennial herbaceous plant from the Rosaceae family, was considered a novel source of obtaining rutin for pharmaceutical purposes. Rutin was extracted from the plant parts collected in the flowering summer period and dried at 40 ± 3 °C. The process was carried out using the ethanol extraction and fractionation of extracted compounds, and it yields the 95 wt% purity crystalline product. The phase composition of the extracted rutin was verified by the XRD analysis and NMR measurements. It was found that 2.85% of rutin could be extracted from *Filipendula ulmaria*, which is 1.2 times higher than the results of similar studies. The biological activity of the isolated rutin was tested on rats. It was established in vivo that the extracted rutin normalizes blood glucose levels (glucose and glycosylated hemoglobin), insulin resistance (HOMA-IR index) and reduces the severity of dystrophic changes in the liver caused by high-fat and high-carbohydrate diets. The introduction of rutin corrects lipid profile indicators (triglycerides, cholesterol, cholesterol fractions in lipoproteins and atherogenic indices), cytolysis indicators of hepatocytes, and liver steatosis (ALT, AST/ALT, triglycerides). Thus, the novel source of rutin opens the possibility for a wide use of this flavonoid in the food technology and pharmaceutical industry.

## 1. Introduction

Due to their species diversity and relatively extensive growth range, plants are important sources of biologically active substances (BAS)—polyphenols, organic acids, vitamins, etc. [1,2,3,4,5,6,7]. Plant extracts are promising raw materials for the production of antimicrobial [8,9,10,11,12,13,14] and antioxidant substances [15,16,17,18,19,20,21] used as part of products that normalize the work of the gastrointestinal tract [22,23,24,25], stimulate the immune system [26], exhibit their anti-cancer [27], cardioprotective properties [28,29] and geroprotective effect [30,31]. Many of the plants are later used in the pharmaceutical industry; for example, *Sophora japonica* L. is used for the industrial production of rutin [32].

Rutin (vitamin P) is a yellow or green–yellow flavonoid, which is a fine crystalline powder [33,34,35]. This BAS exhibits antioxidant [36], anti-inflammatory [37], antitumor [38], neuroprotective [39], cardioprotective [40] and other properties [41], and the compound is actively used for pharmaceutical purposes [42,43]. Since rutin is able to reduce blood glucose levels, prevent the development of insulin resistance, lipid disorders, oxidative stress and normalize blood pressure [44,45], it is included in complexes of biologically active additives used as agents capable of preventing the development of diseases associated with metabolic processes—obesity, diabetes mellitus (Diabetes), cardiovascular diseases (CVD), non-alcoholic fatty liver disease, etc. [6,7,15,26,30,31,32,42,43,44,45,46,47,48,49]. Recently, it has been shown that rutin is able to regulate the microbiota of the gastrointestinal tract, and, hence, the metabolic disorders associated with dysbiosis [49].

In the modern pharmaceutical industry, rutin is commonly obtained from the flower buds of *Sophora japonica* L. (*Styphnolobium japonicum*) [3,50]. However, this raw plant material is distributed only in China, Egypt and Japan, and the process of assembling plant buds is carried out manually, which ultimately leads to a high cost of the final product. Thus, the search for alternative raw plant materials for the effective extraction of rutin on an industrial scale is topical [51]. In several research studies, it was reported that rutin can be extracted from buckwheat (*Fagopyrum esculentum* Moench) [48] and Tatar buckwheat (tartary buckwheat) (*Fagopyrum tataricum* (L.) Gaertn) [52] distributed throughout Eurasia, as well as tall amaranth (*Amaranthus paniculatus* Hutch) grown in many areas of China, India, North and South America and Central Asia [53]. Also, in previous studies, a significant content of rutin was found in *Filipendula ulmaria* [54,55,56], but the technology for extracting rutin from this plant has not yet been presented in the literature.

**Figure 1:**
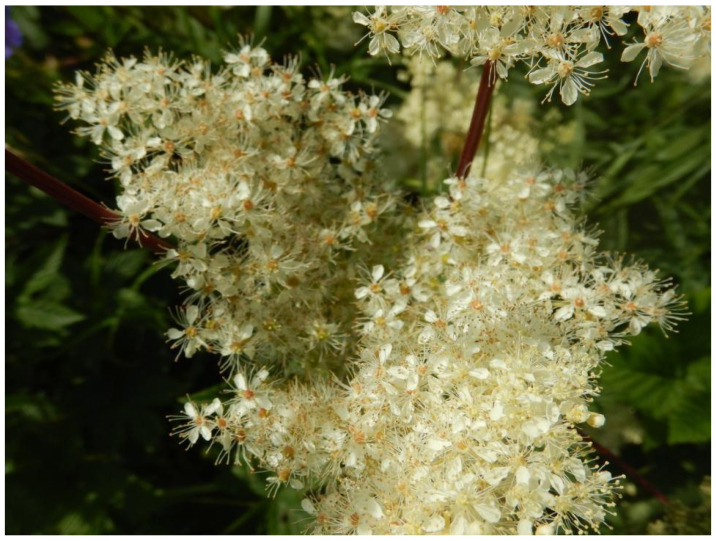
*Filipendula ulmaria* (L.) Photo 409647391, no rights reserved, uploaded by pete_gateley, Source iNaturalist United Kingdom [64].

**Figure 2:**
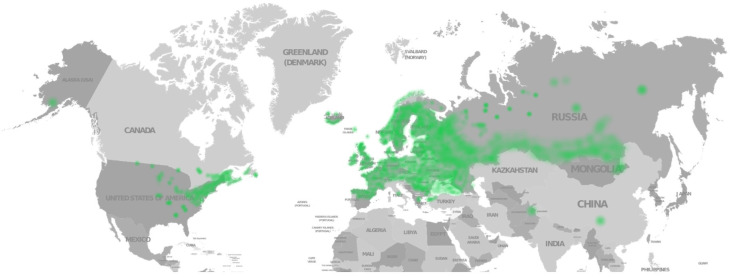
Distribution of *Filipendula ulmaria* (L.) Maxim according to the iNaturalist data [65].

Meadowsweet (*Filipendula ulmaria* (L.) Maxim, Figure 1) is a perennial herbaceous plant from the Rosaceae family, which grows in wetlands, damp meadows, slopes and along water bodies [56,57,58]. The range of meadowsweet covers all of Europe, the Caucasus, Asia Minor and Central Asia, Kazakhstan, Siberia and Mongolia (Figure 2) [59]. In traditional medicine, all parts of the plant are used in the form of tinctures, decoctions, extracts and ointments due to its honey-like aroma and pleasant taste, as well as its anti-inflammatory, antibacterial, antioxidant, anticancer, cytotoxic and other properties for treating diabetes, rheumatism and immune system disorders [56,60,61]. The plant bioactivity is attributed to its content of various polyphenols, organic acids, tannins, essential oils, and other compounds [57,62,63]. Therefore, it can be reasonably assumed that *Filipendula ulmaria* is a promising plant material for extracting BAS, including rutin.

The present study is aimed at the exploration of the potential of *Filipendula ulmaria* as a raw material for the rutin extraction, with the intention of its further use for preventive purposes. In this study, the focus on *Filipendula ulmaria* rather than *Fagopyrum esculentum* and *F. tataricum* is due to the fact that buckwheat fruits are primarily used as a food product for preparing various dishes. In other words, this plant material is mainly used in the food and agricultural industries [66,67,68,69,70]. The technologies for extracting rutin from buckwheat found during a literature review do not allow obtaining a pure substance (purity level less than 85%) [53]. Rutin was extracted from *Filipendula ulmaria* using an original method involving the ethanol extraction and fractionation of the extracted compounds. The biological activity of the isolated rutin was tested on rats.

## 2. Materials and Methods

Herb preparation: A 5–7-year-old *Filipendula ulmaria* plant was obtained from the “Kalina Krasnaya” Nursery (Novosibirsk, Russia). The plant (inflorescences and above-ground parts—where flowers and leaves are included) was collected in the flowering period, from May to June 2023. The collected plant parts were dried at 40 ± 3 °C using a drying oven ShS-80-01 SPU (Smolensk SKTB SPU JSC, Smolensk, Russia) until a constant mass was reached. The dried plant biomass was ground using a laboratory mill LM-202, LLC PLAU (Moscow, Russia). The ground material was sieved through a stainless-steel laboratory sieve TAGLER SLK-200 (LLC Tagler, Moscow, Russia). For the rutin extraction, a powder with a particle size of up to 1 mm was used.

**Figure 3:**
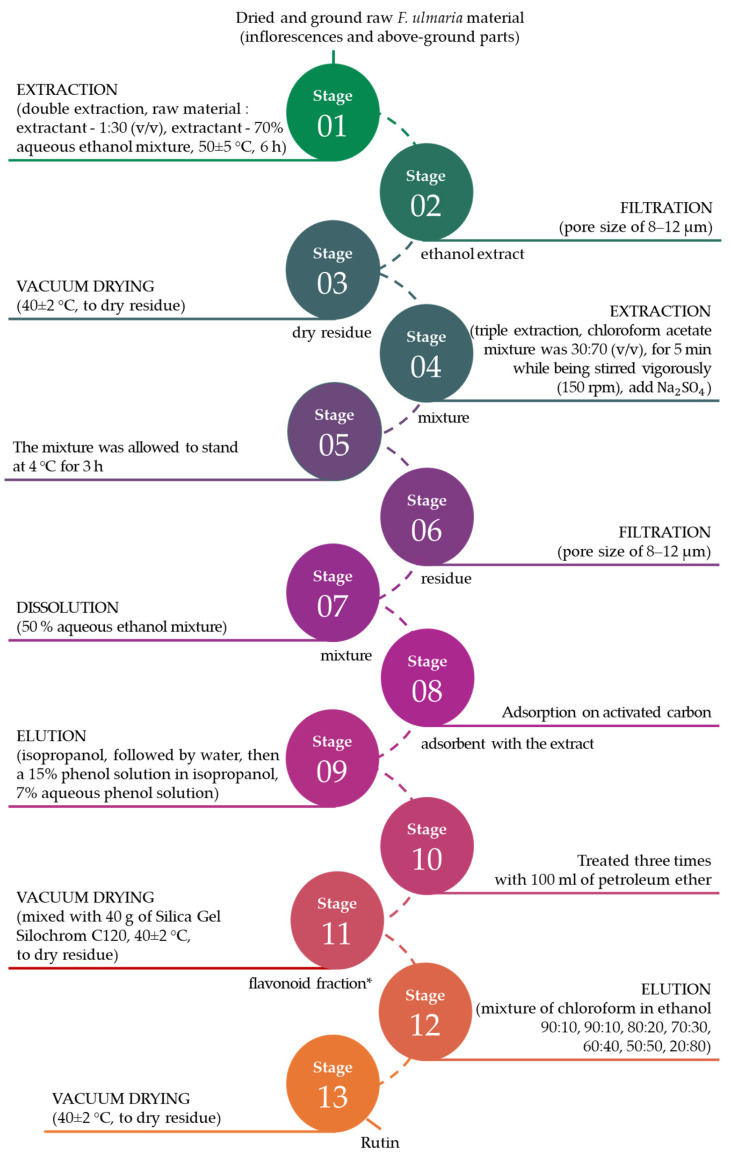
Flowchart of rutin extraction and purification. *—flavonoid fractions, the qualitative and quantitative composition of which was studied by HPLC.

The method for the extraction and purification of rutin developed by the authors is presented in Figure 3.

An LC-20 Prominence chromatographic column (Shimadzu, Kyoto, Japan) was used to assess the qualitative and quantitative composition of the flavonoid fractions. The methodology is presented in the authors’ work [4]. The standards for substances (purity ≥ 99%) of isoquercetin, hyperoside, rutin, spiraeoside, avicularin, and quercetin were acquired by Sigma-Aldrich (Burlington, MA, USA).

**Figure 4:**
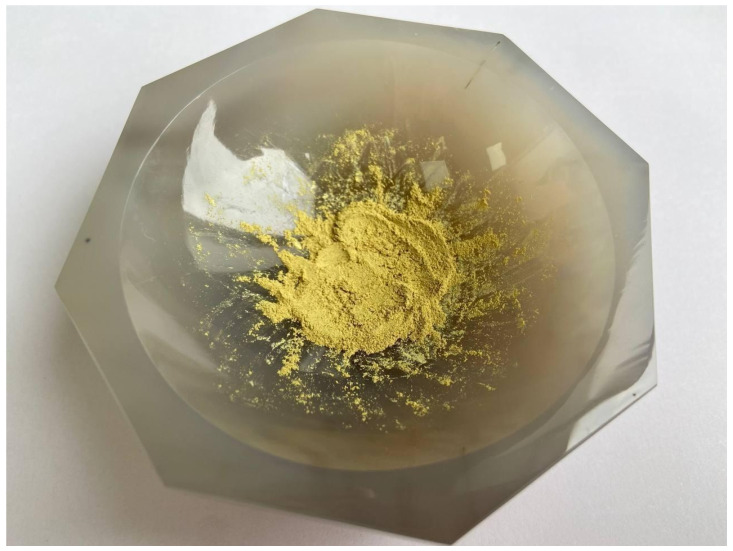
Isolated and purified rutin from *Filipendula ulmaria*.

A photo of rutin isolated from *Filipendula ulmaria*, which is a homogeneous granular yellow powder, is shown in Figure 4. As it was determined by further analysis, the resulting product is rutin with a purity of at least 95%.

The significant features of the method presented by the authors are:

- the elution with chloroform solutions in various ratios, which allowed for the maximum extraction of pure rutin without accompanying impurities and related flavonoids;
- use of an aqueous phenol solution and a phenol solution in isopropanol, ensuring the extraction of ballast substances of a protein-lipid nature;
- use of a rotary evaporator at all stages of evaporation, allowing for the production to be purified from residual solvent amounts.

Reagents: chloroform (substance mass fraction—99.0–99.04%), ethyl acetate (no less than 99%), anhydrous sodium sulfate (no less than 99%), phenol (purchased from JSC LenReaktiv, Saint Petersburg, Russia). Ethanol (96%), isopropanol (no less than 99.7%) and petroleum ether 70–100 were purchased from JSC EKOS-1 (Moscow, Russia).

Rutin identification

For the crystalline structure analysis, a Bruker D8 Advance X-ray diffractometer (Bruker Corporation, Billerica, MA, USA) was used, employing copper radiation (CuKα) with a position-sensitive LynxEye detector, reflection geometry, and rotation. Data collection was conducted using the Bruker DIFFRACplus software kit (Bruker Corporation, Billerica, MA, USA), and analysis was performed using the EVA and Topas V5.0 programs.

For nuclear magnetic resonance (NMR) spectroscopy, 20 mg of the sample was dissolved in 600 μL of dimethyl sulfoxide (DMSO) at a temperature of 25 °C. The resulting solution was placed in a Bruker Avance III HD 500 NMR spectrometer (Bruker Corporation, Billerica, MA, USA) for the registration of NMR spectra. The operating frequencies of the NMR spectrometer are listed in Table 1.

The registration of the ^13^C{^1^H} spectra was performed in a spin-spin interaction suppression mode with ^1^H nuclei (decoupled from ^1^H nuclei) using phase selection (jmod-echo) to separate the signals of CH, CH3/CH2 and C groups in the spectra. To correlate the signals in the ^1^H and ^13^C{^1^H} spectra, additional 2D NMR spectra were obtained: ^1^H-^1^H COSY, ^1^H-^13^C HSQC and ^1^H-^13^C HMBC.

**Figure 5:**
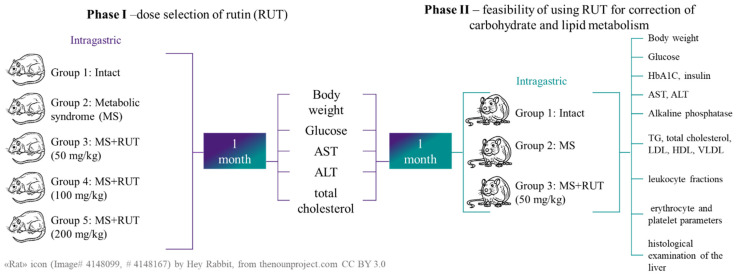
Phases of evaluating the bioactivity of rutin in vivo.

In vivo studies:

The experiment was divided into two stages (Figure 5):

At Phase I, the dose of rutin (RUT) was established (50, 100, 200 mg/kg [71]). The dose of rutin is, on average, 2.69 times less than the recommended dose for an adult.

At Phase II, the effect of the selected rutin dose on the correction of lipid and carbohydrate metabolism was evaluated.

The experiment was conducted on 60 male Wistar rats weighing 190–225 g. The housing conditions and treatment of the animals used in the experiment complied with the recommendations of the International Ethics Committees (EU Council Directive 2010/63/EU). The experimental design was approved by the Ethics Committee of the IIF UB RAS (Protocol No. 03/23, 05/24/2023, Yekaterinburg, Russia). The rats were housed in plastic cages with 5–6 animals per cage, with free access to food and water, and a 12 h light/dark cycle. The intact rats were fed a standard compound feed for laboratory mice and rats, Delta Feeds, LbK 120 C-19 (BioPro company, Novosibirsk, Russia).

The model of metabolic syndrome was induced using a high-fat and high-carbohydrate diet:

56%—standard vivarium feed,

17%—pork fat,

27%—fructose,

10%—fructose solution as the only source of drinking water [67,72].

One month after the start of the experiment, all rats were fed standard vivarium feed, and the rats in groups 3, 4 and 5 were additionally given rutin suspensions intragastrically at doses of 50, 100 and 200 mg/kg, respectively. In all groups, blood samples were taken from the tail vein under ether anesthesia for the biochemical analysis, insulin determination and a complete blood count (CBC). The CBC test was performed using an automated hematology analyzer Celly 70 (Biocode Hycel, Le Rheu, France), designed for animal blood studies in experiments and veterinary practice.

The concentration of glycated hemoglobin (HbA1c) in whole blood was determined using the affinity chromatography method with the GLYCOHEMOTEST reagent kit (LLC Company ELTA, Moscow, Russia). For other biochemical analyses and insulin determination, blood plasma was separated from the cellular components by centrifugation at 1000 g a 4 °C for 10 min. The levels of glucose, total cholesterol (TC), high-density lipoprotein cholesterol (HDL-C) and triglycerides and the activity of AST (aspartate aminotransferase), ALT (alanine aminotransferase) and alkaline phosphatase (ALP) were measured using the Vital Diagnostics reagent kits (JSC Vital Development Corporation, Saint Petersburg, Russia). The AST/ALT ratio (De-Ritis-Quotient) was calculated. The low-density lipoprotein cholesterol (LDL-C) level was determined using the Friedewald formula (Formula (1)):

LDL-C = TC − HDL-C − TG × 0.45, mmol/L,(1)

where:

TC—total cholesterol,

HDL-C—high-density lipoprotein cholesterol,

TG—triglycerides,

TG × 0.45—the cholesterol content in very-low-density lipoproteins (VLDL-C).

The cholesterol atherogenic index (AI) was calculated using Formula (2) [72]:

AI = (TC − HDL-C)/HDL-C(2)

The triglyceride index (TyG) was calculated using Formula (3) [73]:

TyG = ln(Glucose × TG/2)(3)

The optical blood density was determined using an SF-56 LOMO-Spectr spectrophotometer (LLC LOMO-Microsystems, Saint Petersburg, Russia). The serum insulin level was measured using the Rat Insulin ELISA kit (Thermo Fisher Scientific, Waltham, MA, USA) and the Lazurit immunoassay analyzer (Dynex Technologies, Chantilly, VA, USA). The insulin resistance was assessed using the HOMA-IR index, Formula (4) [74]:

HOMA-IR = fasting insulin (μU/mL) × fasting glucose (mmol/L)/22.5(4)

The animals were euthanized by the intramuscular injection of xylazine (TLC Pharmaceutical Standards, Newmarket, ON, Canada) at a dose of 0.1 mL/100 g of rat body weight, followed by the administration of Zoletil (TLC Pharmaceutical Standards, Newmarket, ON, Canada) at a dose of 5 mg/kg body weight after 15 min.

Following euthanasia and median laparotomy, the liver was removed from the animals. Their tissue fragments were fixed in a 10% aqueous solution of neutral formalin for 24 h. After 8 h of washing, the material underwent the standard histological processing in an automated tissue processor, Leica TP 1020 (Leica Microsystems, Wetzlar, Germany). Sections were prepared on a Leica SM 2000R microtome (Leica Microsystems, Wetzlar, Germany) with a thickness of 3–4 μm. The immunohistochemical examination of the liver was performed on paraffin sections.

The statistical analysis of the material was conducted using the Statistica 6.0 software (Stat. Soft Inc., Tulsa, OK, USA). The data were presented as mean ± standard error of the mean. Due to the small sample size, the method of non-parametric statistics was applied. The statistical significance of differences in the compared independent samples was evaluated using the Mann–Whitney U test. A significant level of 5% (*p* < 0.05) was used when testing statistical hypotheses.

Phase I. Rutin dose selection

In the first stage of the experiment, 30 rats were used. The rats were divided into 5 groups, with 6 animals per group:

Group 1—intact group (control group);

Group 2—metabolic syndrome (MS);

Group 3—MS + RUT 50 mg/kg;

Group 4—MS + RUT 100 mg/kg;

Group 5—MS + RUT 200 mg/kg.

One month after the start of the experiment, all the rats received standard vivarium feed, and the rats in groups 3, 4 and 5 were additionally administered the rutin suspension intragastrically at doses of 50, 100 and 200 mg/kg, respectively. The animal body weight was measured: M1—before the experiment, M2—after 1 month and M3—at the end of the experiment. The biochemical indicators of glucose levels, cholesterol and aminotransferase activity (AST, ALT) were evaluated.

Phase II. Evaluation of the feasibility of using rutin at a dose of 50 mg/kg to correct carbohydrate and lipid metabolism indicators in rats with metabolic syndrome

A total of 30 rats were used, and the experiment involved forming three study groups:

Group 1—intact group (control group);

Group 2—metabolic syndrome;

Group 3—MS + RUT 50 mg/kg.

The study followed a design similar to the one described above. For these groups, a histological examination of the liver was conducted.

## 3. Results

Results of isolation, analysis, and purification of rutin from its derivatives

**Figure 6:**
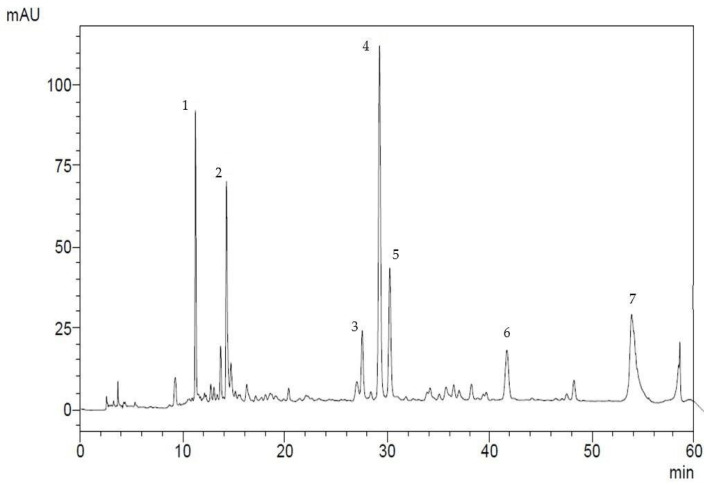
Chromatogram showing the content of flavonoid fraction in the *Filipendula ulmaria*: 1, 2—isoquercetin, 3—hyperoside, 4—rutin, 5—spiraeoside, 6—avicularin, 7—quercetin.

The composition of the obtained flavonoid fraction from *Filipendula ulmaria* is shown in Figure 6 and Table 2.

Chromatographic analysis confirmed the presence of rutin in the obtained flavonoid fraction. The total flavonoid content was 4.75%. Subsequently, the rutin fraction was purified from its derivatives.

**Figure 7:**
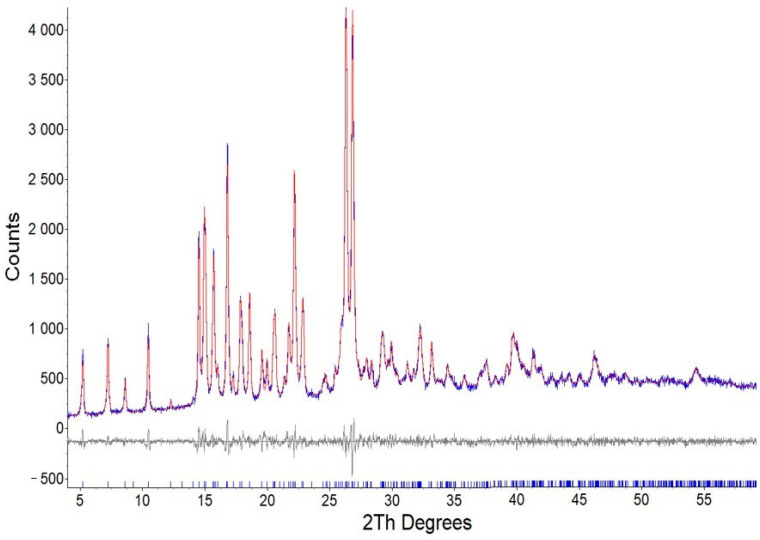
XRD pattern of the rutin sample (blue curve) and the theoretical diffractogram calculated for its cell (red line), along with its difference curve (gray line). The vertical ticks indicate the calculated peak positions.

The results of the XRD analysis indicated that the rutin sample is crystalline and monophase, as seen in Figure 7; more detailed results are presented in Appendix A (Table A1).

**Figure 8:**
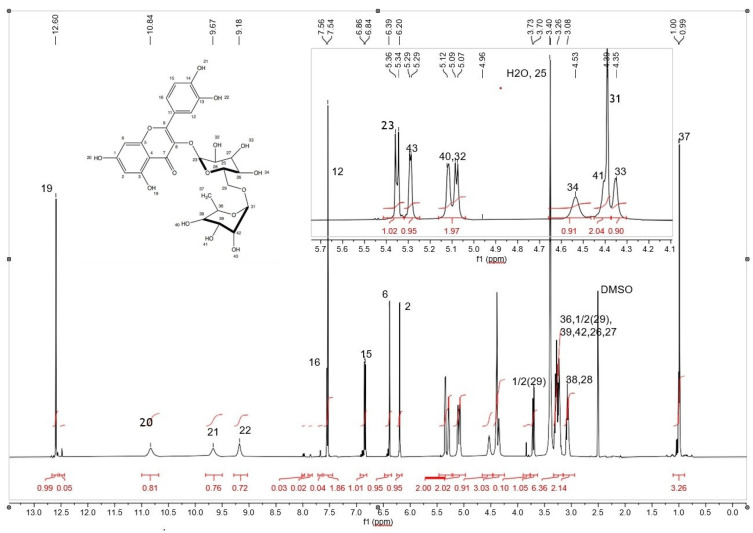
NMR spectrum of the rutin sample (^1^H).

**Figure A1:**
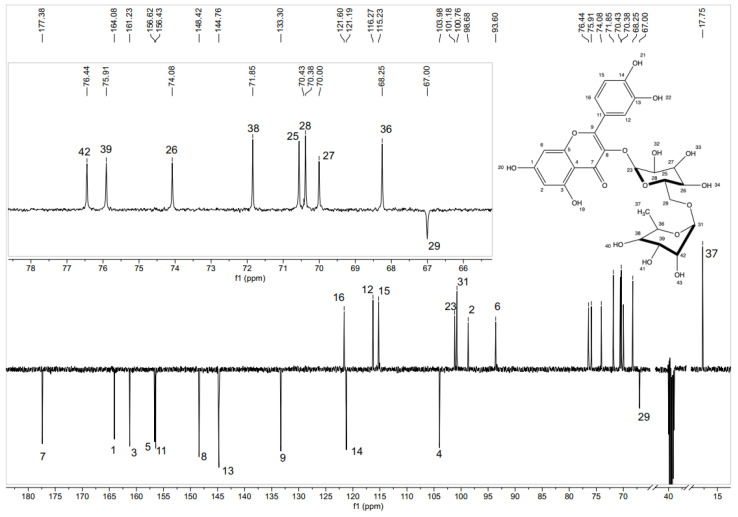
^13^C{^1^H} NMR spectrum of the rutin sample.

**Figure A2:**
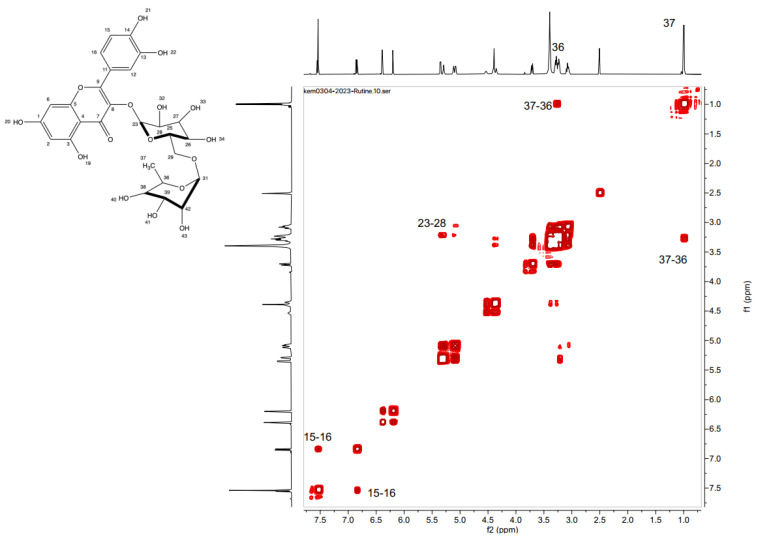
2D ^1^H^1^H–COSY NMR spectrum of the rutin sample.

**Figure A3:**
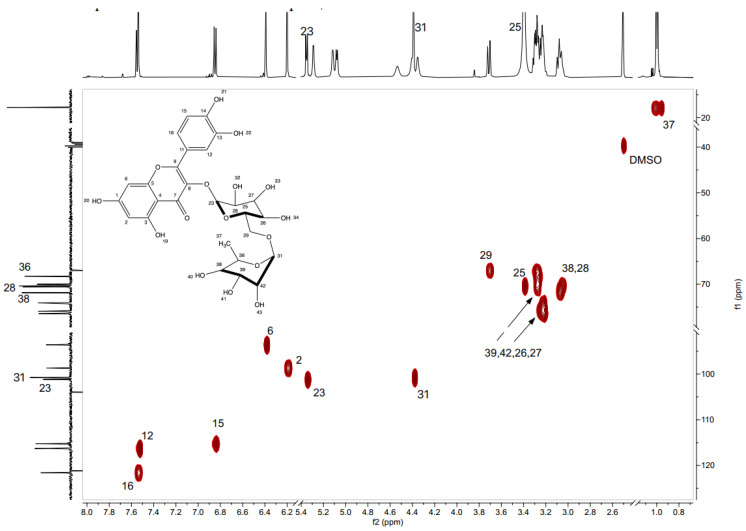
^1^H^13^C-HSQC NMR spectrum of the rutin sample.

**Figure A4:**
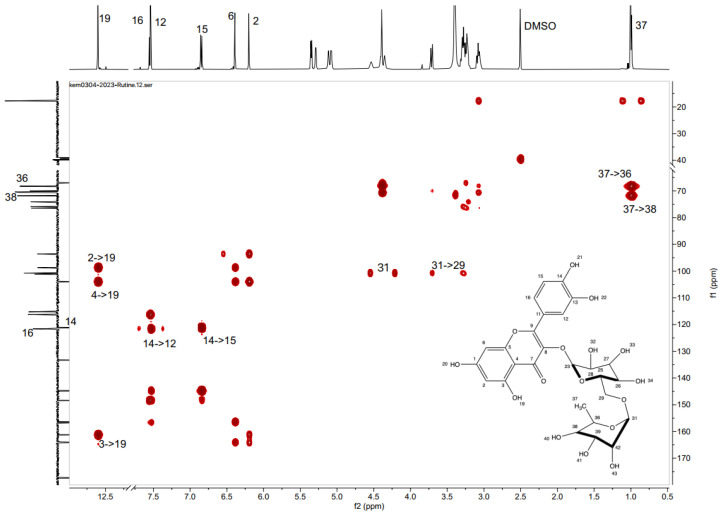
^1^H^13^C-HMBC NMR spectrum of the rutin sample.

Based on the results of the XRD analysis, it was determined that the substance crystallizes in a monoclinic cell (space group *P*2_1_) with cell parameters *a* = 16.9495(8) Å, *b* = 6.7822(4) Å, *c* = 12.2373(8) Å, β = 94.367(4)°, *V* = 1402.66(14) Å^3^, *R*-Bragg = 0.252%, *R*exp = 4.3%, *R*wp = 5.59%, *R*p = 4.26%, GOF = 1.29%. In Figure 8, the NMR spectrum of the rutin sample is shown, and more detailed results are reflected in Appendix A in Figure A1, Figure A2, Figure A3 and Figure A4.

It was found that the NMR spectra of the presented sample correspond to the target product—rutin.

Impurity signals are absent. Based on the number of signals, their positions in the spectra (chemical shifts), integral intensities, their ratios, signal multiplicities and spin-spin coupling constants (J values), the obtained spectra match the target product. The assignment of signals is presented in Appendix A in Table A2 (^1^H spectrum) and Table A3 (^13^C{^1^H} spectrum).

During the purification process, it was possible to obtain 23.5 g of flavonoids from 500 g of plant material, of which 14.25 g was rutin.

Results of the in vivo study

Phase I

To investigate the effect of rutin on the body weight changes in rats with metabolic syndrome, rutin was administered intragastrically to three groups of rats. In Table 3 are the changes in the rats’ body weight.

During the experiment, a significant increase in the body weight of the rats (M2) and weight gain (M2–M1) was observed one month after the start of the experiment and modeling of the metabolic syndrome in groups 2–5, compared to the indicators of intact rats. In the following month, the body weight (M3) of group 2, which did not receive rutin, is not increased compared to M2; however, the weight gain compared to the baseline (M3–M1) remains significantly higher than that of the intact rats. In groups 3–5, which received different doses of rutin for one month, the values of M3 were somewhat lower than M2 in the same groups, but the differences were insignificant. At the same time, the weight gain over the entire period of the experiment (M3–M1) in groups 3–5 was no greater than in the intact rats, unlike (M3–M1) in group 2. No significant differences in weight and weight gain were found between groups 3, 4 and 5. Thus, modeling the metabolic syndrome contributed to the accumulation of body weight in the rats during the first month of the experiment, while the subsequent administration of rutin, regardless of the dose, prevented the excessive weight gain to a greater extent than the discontinuation of the high-fat and high-carbohydrate diet. In Table 4 are the biochemical indicators of glucose, cholesterol levels and aminotransferase activity in the rats’ blood.

When comparing glucose levels, it can be noted that the modeling of metabolic syndrome without the administration of rutin (Group 2) is accompanied by a slight but significant increase in glucose levels. In contrast, the rats that received rutin (Groups 3, 4, and 5) showed normalized glucose levels, which were significantly different from the glucose levels in Group 2. The total cholesterol levels in rats with MS are also significantly increased compared to the intact group and the rats that were administered rutin, i.e., in Groups 3, 4 and 5. No significant differences were found in the AST activity across all animal groups; however, in Groups 2, 4 and 5, the ALT activity is increased, and the AST/ALT ratio is decreased compared to the intact rats. This indicates the presence of cytolysis primarily in the liver. The changes in the ALT activity and the AST/ALT ratio in Group 2 are likely associated with the manifestation of steatosis, a common complication of the metabolic syndrome.

In Groups 4 and 5, which received rutin at doses of 100 and 200 mg/kg after modeling MS, the changes in the liver profile parameters may be linked to the excessive doses of rutin. As a xenobiotic, rutin may be metabolized in the liver through microsomal oxidation reactions, leading to the formation of reactive oxygen species that can damage hepatocyte membranes. It is noted that flavonoids can exert not only antioxidant but also pro-oxidant effects, especially at excessive doses. Thus, all three investigated doses of rutin—50, 100 and 200 mg/kg—equally contributed to lowering glucose and cholesterol levels in rats with the metabolic syndrome. The dose of 50 mg/kg prevented the hepatocyte cytolysis, while the doses of 100 and 200 mg/kg likely represented an excessive burden on the liver detoxification systems. As a result, the dose of 50 mg/kg can be considered as optimal for correcting carbohydrate and lipid metabolism indicators in rats with the metabolic syndrome and will be used in the second stage of the study.

Phase II

During the experiment, it was found that the changes in the body mass of the rats with the metabolic syndrome are correspondent to the changes established in the first stage of the experiment: the mass (M2) and the increase in mass (M2–M1) were higher compared to the same indicators in the intact group after one month from the start of the experiment. In the following month, there was no accumulation of mass, but the difference between M3 and (M3–M1), compared to the norm, was significant, as is shown in Table 5.

In the rats with metabolic syndrome that received rutin (Group 3), the increase in mass before the treatment (M1) was not different from the value in Group 2. After administering rutin at a dose of 50 mg/kg for 1 month, the M3 value was normalized, and the increase in mass (M3–M1) was significantly lower than that of the animals in Group 2. The modeling of MS in the second phase of the experiment, as in the first phase, induced a state of hyperglycemia. A significant increase in glucose and glycosylated hemoglobin levels was observed in Group 2 rats, compared to the intact Group 1 (Table 6). The accumulation of HbA1C in rats is a sign of persistent hyperglycemia lasting at least one month.

A slight increase in insulin levels in the rats with the metabolic syndrome, compared to the intact group, did not reach statistical significance; however, an elevated HOMA-IR index was observed, indicating the development of insulin resistance. In assessing the risk of developing diabetes with glucose levels below 7 mmol/L, HOMA-IR is more informative than glucose or fasting insulin levels alone. For instance, in rats, 30 days after modeling type 2 diabetes with nicotinamide-streptozotocin, the HOMA-IR index increased, while the glucose tolerance decreased.

The administration of rutin at the dose of 50 mg/kg to the rats with the metabolic syndrome contributed to the normalization of glucose levels, HbA1C and HOMA-IR, with all three indicators significantly different from the glycemic levels in the rats with the metabolic syndrome (Group 2). Modeling the metabolic syndrome did not affect the AST activity, but was accompanied by increased ALT activity and a decrease in the AST/ALT ratio, confirming the development of a cytolytic syndrome in the liver due to the effects of a high-fat and high-carbohydrate diet in the course of a month. At the same time, the alkaline phosphatase (ALP) activity in the rats with the metabolic syndrome did not increase, indicating the absence of a cholestatic syndrome. After the administration of rutin to the rats in Group 3, the ALT activity and the De Ritis ratio returned to levels similar to those of intact rats, and these parameters were significantly different from those of the untreated animals. In the study of lipid metabolism indicators (Table 7), an increase in triglyceride (TG) levels was observed in the rats with the metabolic syndrome (Group 2), which correlates with the increase in the ALT and the decrease in the AST/ALT ratio, as elevated TG levels in fasting plasma are a sign of the steatosis formation in the liver.

In the rats with the metabolic syndrome (Group 2), a significant increase in total cholesterol, cholesterol of atherogenic fractions (LDL and VLDL) and a decrease in cholesterol of the anti-atherogenic fraction (HDL) were also observed. This led to an increase in the atherogenic coefficients for cholesterol (AI) and triglycerides (TyG). The triglyceride index (TyG) is used as an indicator reflecting the insulin resistance and the risk of developing cardiovascular diseases, which is also relevant for diagnosing diabetes mellitus [75,76]. The levels of total cholesterol and atherogenic coefficients in the rats with the metabolic syndrome are insufficient to cause macroangiopathy. In humans, macroangiopathy develops if plasma cholesterol levels exceed 5 mmol/L and the cholesterol atherogenic index is above 2.8 [68,72].

When examining hematological parameters, no significant differences were found between the metabolic syndrome groups (Group 2) and the intact group (Group 1), as well as between the metabolic syndrome + rutin group (Group 3), in terms of the total leukocyte count and absolute and relative values of leukocyte fractions (Table 8).

The obtained data indicate the absence of a chronic inflammatory process involving lymphocytes and monocytes, as well as an acute inflammatory process involving granulocytes at the organismal level during the modeling of metabolic syndrome. However, this does not rule out the development of localized inflammatory processes in the adipose tissue and the liver. The administration of rutin to rats with MS did not affect the total number of leukocytes or their fractions. The use of a high-fat and high-carbohydrate diet was accompanied by a significant increase in total hemoglobin, hemoglobin content in individual erythrocytes (MCH) and hemoglobin concentration in individual erythrocytes (MCHC), compared to the indicators of the intact group (Table 9).

**Figure 9:**
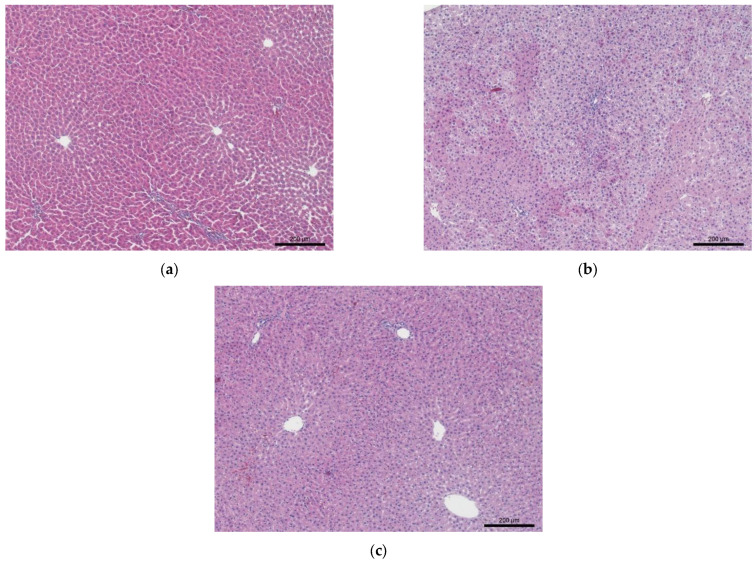
Representative microphotographs of the liver from rats in the experimental groups. Staining is with hematoxylin and eosin. Magnification 10×: (**a**) intact animals; (**b**) animals with the metabolic syndrome; (**c**) animals with the metabolic syndrome treated with 50 mg/kg rutin.

**Figure 10:**
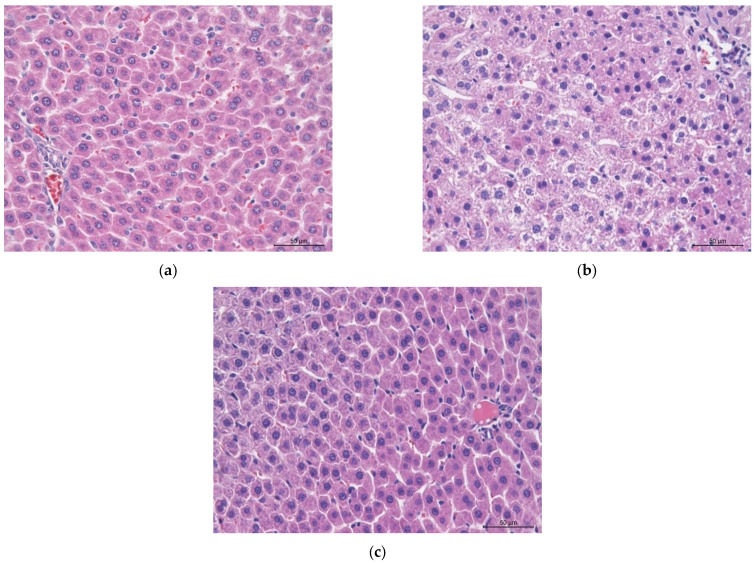
Representative microphotographs of the liver from rats in the experimental groups. Staining is with hematoxylin and eosin. Magnification 40×: (**a**) intact animals; (**b**) animals with the metabolic syndrome; (**c**) animals with the metabolic syndrome treated with 50 mg/kg rutin.

At the same time, the number of erythrocytes, hematocrit, erythrocyte volume (MCV) and erythrocyte distribution width (RDW) in the metabolic syndrome group (Group 2) remained within normal limits. The increase in hemoglobin levels during the MS modeling may be due to the compensation for hypoxia, which is one of the chronic complications of type 2 diabetes, aligning with the experiment findings of accumulated glycated hemoglobin, which is unable to transport oxygen (Table 6). After the administration of rutin, the total hemoglobin, MCV and MCHC levels remained higher than in the intact group. It is possible that more time is needed to correct these indicators, as the lifespan of rat erythrocytes is 37 days, and the renewal of erythrocytes with elevated hemoglobin content has not yet been completed [77]. No significant changes were found in the number of platelets and platelet indices (MPV—platelet volume, PDW—platelet distribution width, and Pct—plateletcrit) in either the MS group (Group 2) or the MS + rutin group (Group 3), compared to the indicators of the intact animals. The results of the histological examination of the liver in the experimental groups of rats (Groups 1–3) are shown in Figure 9 and Figure 10.

In animals (Group 1), the histological structure of the liver fully corresponds to the norm, characterized by a lobular structure (Figure 9a and Figure 10a). Sinusoidal spaces are located between rows of hepatocytes. The hepatocytes have a polygonal shape and are arranged in rows, radiating from the periphery of the lobule towards the central veins. The cytoplasm of the hepatocytes is oxyphilic, containing lightened areas (glycogen inclusions dissolved during the histological processing of the material). The nuclei of hepatocytes are typically slightly polymorphic, with clearly defined nucleoli and chromatin clumps, and binucleated cells are often found.

In the rats with the metabolic syndrome (Group 2), the lobular structure of the liver is preserved (Figure 9b and Figure 10b). The central veins and sinusoids are not expanded. Hepatocytes exhibit greater polymorphism than normal, with areas of uneven staining, showing sharply oxyphilic cytoplasm. At the same time, the cytoplasm of most hepatocytes is markedly granular with clumps of basophilic inclusions and vacuolization. An increase in the nuclear polymorphism of the hepatocytes is noted. The nuclei remain centrally located, the karyolemma is intact, and nucleoli are visible. These signs indicate the development of dystrophic processes in the organ, suggesting the formation of diffuse fine-grained fatty degeneration. There are no signs of liver fibrosis.

In the group with the metabolic syndrome + 50 mg/kg rutin (Group 3), the histoarchitecture of the liver corresponds to normal, with the lobular structure preserved (Figure 9c and Figure 10c). In particular, moderate expansion of the central veins is observed, while sinusoidal capillaries are not expanded. Hepatocytes exhibit greater polymorphism than normal but less than in the metabolic syndrome group (Group 2). On average, the cytoplasm of hepatocytes is characterized by moderate granularity and vacuolization. The severity of pathomorphological changes varies among the rats; some exhibit moderate dystrophic changes in the liver, while others show more pronounced alterations. Nuclear polymorphism is lower than in the metabolic syndrome group (Group 2). The nucleus is centrally located, the karyolemma is intact, and nucleoli are visible. No fibrotic changes are detected in the liver. Microscopic signs indicate a reduction in the severity of pathological changes in the liver, compared to the metabolic syndrome group (Group 2).

## 4. Discussion

The aim of this study was to investigate the potential of *Filipendula ulmaria* as a source of rutin extraction for further use in preventive medicine. The results obtained showed that the extraction and purification method proposed by the authors allows for the recovery of a 23.75 g flavonoid fraction from 500 g *Filipendula ulmaria*, of which 14.25 g is rutin (with a purity of at least 95%). As a result of this work, it was found that the use of *Filipendula ulmaria* (inflorescences and above-ground parts) for the extraction of rutin is promising. These results are consistent with the scientific literature.

A method for obtaining a total flavonoid extract from *Filipendula ulmaria* was proposed earlier [78]. The method involves a single extraction of air-dried raw material using an organic solvent—40% aqueous ethanol is used as the extracting agent, with a raw material to solvent ratio of 1:50. The extraction is carried out in a boiling water bath for 90 min. A disadvantage of this method is the low level of extract purification, and the obtained extract contains unseparated flavonoids. Also, the method for obtaining rutin from the vegetative mass of buckwheat (*Fagopyrum esculentum*) was reported [48], and it includes the following stages: the ground plant biomass is defatted with 100% acetone, then extracted for the first time with a 70% aqueous ethanol solution (raw material to solvent ratio of 1:10) in a boiling water bath for 60 min. The extract is separated from the residue. Then, the residue is extracted a second time with a 70% aqueous ethanol solution (ratio 1:5) at room temperature for 60 min. The extract is again separated from the residue. The extracts are combined, concentrated under vacuum and dried. The rutin residue is transferred to a funnel with cotton wool and treated twice with 40 mL of diethyl ether, ethyl acetate and then extracted with 20 mL of butanol. The disadvantages of this method are the long time and labor intensity of the process, as well as the low purity of the obtained rutin. Additionally, the method insufficiently describes the parameters of the purification process and relies solely on the recrystallization and filtration through paper filters for the purification of rutin. In the presented method, 13.86 g of rutin was extracted from 500 g of plant material.

The method of extracting rutin from the aerial parts of amaranth (*Amaranthus paniculatus*) is the closest to the extraction method proposed in the present study. This method involves the extraction of dried aerial parts (raw material to solvent ratio of 1:10) using a 70% aqueous ethanol solution, heated for 1 h, followed by the treatment of the extract with ethyl acetate and recrystallization of the residue from water. In this case, the purity rate of extracted rutin was as high as 85%. A disadvantage of the method proposed is the lack of information regarding the extraction temperature; from 500 g of plant material, the yield of rutin was 10.4 g [53].

In this study, we investigated the ability of isolated rutin to correct levels of lipid and carbohydrate metabolism in vivo (in an experimental model of metabolic syndrome in rats). Metabolic syndrome in rats was modeled using a high-fat and high-carbohydrate diet. Atherosclerosis is not a characteristic pathological condition for rodents, but the changes in lipid metabolism indicators toward an atherogenic situation are consistent across all vertebrate animals and humans. The changes in total cholesterol and lipoprotein fractions observed in the rats with the metabolic syndrome confirm alterations in lipid metabolism, and that indicates a potential progression toward atherosclerosis. These changes are sufficient to warrant the study of lipid metabolism correction in rats. Administering rutin at a dose of 50 mg/kg to the rats with metabolic syndrome for one month helped normalize all investigated lipid metabolism indicators, and these values significantly differed from those of rats that did not receive rutin.

The results obtained are consistent with the data in the literature. Indeed, in the study by S. Li and co-authors [43], it was established that the administration of rutin (in the form of sodium salt to increase its water solubility and bioavailability) in drinking water (0.2 mg/mL) for 8 months increased energy expenditure in mice—mice burned more fat than those in the control groups (which did not receive rutin); it also increased the expression of genes related to the lipid metabolism in the liver—*Far1, Steap4, Il1rn, Isyna1, Lcn2, Enho, Angptl8*—and affected the concentration of lipid metabolites, reducing triglyceride levels. In the study by C. Cai and co-authors [48], rutin (100 and 200 mg/kg body weight), isolated from tartary buckwheat (*Fagopyrum tataricum*), reduced glucose levels and increased the total cholesterol concentration; it stimulated the growth of representatives of the normal gastrointestinal microbiota that produce short-chain fatty acids and decreased the abundance of microbiota associated with the development of diabetes—*Escherichia* and *Mucispirillum*—in C57BL/6J mice, in which type 2 diabetes was induced. In the study by J. Yang [77], it was found that a 7-week administration of a deglycosylated rutin solution (100 mg/kg body weight, solvent 0.1% DMSO) orally to male C57BL/6J mice, which had obesity induced by a high-fat diet, resulted in a 33% reduction in their body weight compared to the control group and improved lipid metabolism parameters (levels of total cholesterol, triglycerides and LDL).

## 5. Conclusions

It was established in this study that it is advisable to use *Filipendula ulmaria*, which grows relatively widely across Eurasia, as a source for extracting rutin. The technology was developed for the extraction and purification of the bioactive substance from *Filipendula ulmaria*, and it allows for the average yield of flavonoid fractions to be 4.75% from the plant, of which 2.85% is rutin with a purity of at least 95%. These indicators are better by an average of 1.2 times than those of analogs—considered by the authors—extractions from *Fagopyrum esculentum* and *Amaranthus paniculatus*.

Thus, an expansion of the raw material base for sources of rutin used in food and pharmaceutical industries was predicted in this study. It was established in vivo that the extracted rutin normalizes blood glucose levels (glucose and glycosylated hemoglobin), insulin resistance (HOMA-IR index) and reduces the severity of dystrophic changes in the liver caused by high-fat and high-carbohydrate diets. The introduction of rutin corrects lipid profile indicators (triglycerides, cholesterol, cholesterol fractions in lipoproteins and atherogenic indices), cytolysis indicators of hepatocytes and liver steatosis (ALT, AST/ALT, triglycerides). The administration of rutin prevents excessive weight gain to a greater extent than the cessation of a high-fat and high-carbohydrate diet. Accordingly, the results obtained are consistent with the scientific data on the benefits of rutin.

A limitation of this study is that the developed technology for extracting rutin from *Filipendula ulmaria* was not compared with the extraction technology from the reference plant *Sophora japonica* L. (due to the inability to obtain this plant in the natural state). In the future, the research should be focused on studying the ability to reduce the body mass index in people suffering from obesity through the systematic intake of rutin and/or rutin as a part of various dietary supplements.

## Figures and Tables

**Table 1:** Operating frequencies used in the NMR study.

Nucleus	Operating Frequency, MHz
^1^H	500.13
^13^C	125.74

**Table 2:** Flavonoid fraction content in the *Filipendula ulmaria*.

Spike Number	BAS	Retention Time, min	Content, %
1	Isoquercetin	11.24 ± 0.1	0.61 ± 0.0015
2		14.1 ± 0.2	0.37 ± 0.0009
3	Hyperoside	27.2 ± 0.3	0.16 ± 0.0012
4	Rutin	29.1 ± 0.1	2.85 ± 0.0011
5	Spiraeoside	30.2 ± 0.4	0.34 ± 0.0021
6	Avicularin	42.0 ± 0.2	0.20 ± 0.0007
7	Quercetin	53.0 ± 0.1	0.22 ± 0.0003

**Table 3:** Changes in the rats’ body weight during the experiment (Phase I).

Indicator	Group 1 (n = 6)	Group 2 (n = 6)	Group 3 (n = 6)	Group 4 (n = 6)	Group 5 (n = 6)
Weight before the experiment (M1), g	213.3 ± 8.0	216.7 ± 6.8	230.0 ± 8.2	219.2 ± 5.2	215.0 ± 4.5
Weight after 1 month (M2), g	227.5 ± 8.9	258.3 ± 8.9 *	270.0 ± 5.6 *	260.0 ± 7.7 *	256.7 ± 3.8 *
(M2–M1), g	14.2 ± 2.0	41.7 ± 3.3 *	40.0 ± 3.4 *	40.8 ± 6.1 *	42.5 ± 2.8 *
Weight at the end of the experiment (M3), g	230.8 ± 8.8	257.5 ± 8.0	257.5 ± 6.8	248.3 ± 7.4	244.2 ± 5.8
(M3–M1), g	17.5 ± 3.1	40.8 ± 5.4 *	27.5 ± 4.8	29.2 ± 5.8	29.2 ± 4.0

*—the difference from the indicator of the intact animals group is statistically significant at *p* < 0.05.

**Table 4:** Biochemical indicators of rats in groups 1–5 during the first phase of the experiment.

Indicator	Group 1 (n = 6)	Group 2 (n = 6)	Group 3 (n = 6)	Group 4 (n = 6)	Group 5 (n = 6)
Glucose, mmol/L	5.39 ± 0.28	7.04 ± 0.22 *	5.81 ± 0.18 ^#^	5.93 ± 0.28 ^#^	5.70 ± 0.21 ^#^
AST, U/L	15.3 ± 0.5	15.2 ± 0.6	15.4 ± 0.4	15.6 ± 0.7	15.5 ± 0.8
ALT, U/L	11.4 ± 0.4	15.2 ± 0.9 *	13.7 ± 1.0	16.2 ± 1.0 *	16.6 ± 1.9 *
AST/ALT	1.36 ± 0.07	1.01 ± 0.03 *	1.15 ± 0.09	0.99 ± 0.08 *	0.97 ± 0.06 *
TC, mg/dL	1.90 ± 0.05	2.77 ± 0.18 *	1.81 ± 0.09 ^#^	2.06 ± 0.07 ^#^	2.04 ± 0.12 ^#^

*—difference from the intact group is significant at *p* < 0.05; ^#^—difference from group 2 is significant at *p* < 0.05.

**Table 5:** Changes in the body mass of rats in the experiment (Phase II).

Indicator	Group 1 (n = 10)	Group 2 (n = 10)	Group 3 (n = 10)
Mass before the experiment (M1), g	217.0 ± 4.8	217.0 ± 3.7	218.5 ± 4.2
Mass after 1 month (M2), g	232.0 ± 4.1	258.0 ± 4.4 *	261.0 ± 5.7 *
(M2–M1), g	15.0 ± 1.5	41.0 ± 4.1 *	42.5 ± 2.5 *
Mass at the end of the experiment (M3), g	236.0 ± 3.6	259.5 ± 4.2 *	249.0 ± 4.5
(M3–M1), g	19.0 ± 2.1	42.5 ± 3.7 *	30.5 ± 2.4 * ^#^

*—difference from the intact group is significant at *p* < 0.05; ^#^—difference from Group 2 is significant at *p* < 0.05.

**Table 6:** Glycemic indicators and organ conditions in rats during the second stage of the experiment.

Indicator	Group 1 (n = 10)	Group 2 (n = 10)	Group 3 (n = 10)
Glucose, mmol/L	5.31 ± 0.16	7.12 ± 0.22 *	5.69 ± 0.15 ^#^
HbA1C, %	4.3 ± 0.1	5.2 ± 0.1 *	4.6 ± 0.1 ^#^
Insulin, μU/mL	2.17 ± 0.14	2.69 ± 0.22	2.48 ± 0.28
HOMA-IR	0.516 ± 0.040	0.852 ± 0.071 *	0.635 ± 0.075
AST, U/L	15.2 ± 0.2	15.2 ± 0.3	14.9 ± 0.4
ALT, U/L	11.5 ± 0.3	15.1 ± 0.4 *	11.9 ± 0.4 ^#^
AST/ALT	1.33 ± 0.04	1.01 ± 0.02 *	1.27 ± 0.06 ^#^
ALP, U/L	59.1 ± 2.4	61.2 ± 2.2	59.8 ± 2.4

*—difference from the intact group is statistically significant at *p* < 0.05; ^#^—difference from Group 2 is statistically significant at *p* < 0.05.

**Table 7:** Lipid metabolism indicators (Phase II).

Indicator	Group 1 (n = 10)	Group 2 (n = 10)	Group 3 (n = 10)
TG, mmol/L	0.361 ± 0.017	0.716 ± 0.020 *	0.319 ± 0.014 ^#^
TC, mmol/L	1.878 ± 0.046	2.807 ± 0.047 *	1.868 ± 0.029 ^#^
HDL-C, mmol/L	1.400 ± 0.061	0.945 ± 0.016 *	1.427 ± 0.023 ^#^
LDL-C, mmol/L	0.314 ± 0.039	1.550 ± 0.042 *	0.293 ± 0.021 ^#^
VLDL-C, mmol/L	0.164 ± 0.008	0.310 ± 0.015 *	0.148 ± 0.006 ^#^
AI	0.358 ± 0.048	1.974 ± 0.057 *	0.310 ± 0.017 ^#^
TyG	0.955 ± 0.048	2.462 ± 0.074 *	0.911 ± 0.054 ^#^

*—difference from the indicator of the intact animal group is significant at *p* < 0.05; ^#^—difference from the indicator of Group 2 is significant at *p* < 0.05.

**Table 8:** Total leukocyte count and leukocyte fractions in rats during Phase II of the experiment.

Indicator	Group 1 (n = 10)	Group 2 (n = 10)	Group 3 (n = 10)
Leukocytes, thou/mcL	6.73 ± 0.39	7.64 ± 0.45	7.51 ± 0.57
Lymphocytes, thou/mcL	4.49 ± 0.35	5.09 ± 0.34	5.29 ± 0.49
Monocytes, thou/mcL	0.21 ± 0.02	0.21 ± 0.02	0.22 ± 0.02
Granulocytes, thou/mcL	2.03 ± 0.17	2.34 ± 0.12	2.00 ± 0.10
Lymphocytes, %	66.2 ± 2.6	66.0 ± 1.3	69.4 ± 1.6
Monocytes, %	3.3 ± 0.2	3.1 ± 0.1	2.9 ± 0.1
Granulocytes, %	30.5 ± 2.6	30.9 ± 1.2	27.6 ± 1.6

**Table 9:** Erythrocyte and platelet indicators (Phase II).

Indicator	Group 1 (n = 10)	Group 2 (n = 10)	Group 3 (n = 10)
Red blood cell count, million/μL	8.47 ± 0.08	8.57 ± 0.12	8.67 ± 0.06
HbA1C, g/dL	149.7 ± 2.0	162.4 ± 2.0 *	161.4 ± 1.3 *
Hct, %	45.1 ± 0.7	45.6 ± 0.5	46.0 ± 0.4
Mean corpuscular volume (MCV), fl	53.3 ± 0.8	53.2 ± 0.4	53.1 ± 0.4
Mean corpuscular hemoglobin (MCH), pg	17.7 ± 0.3	18.9 ± 0.2 *	18.6 ± 0.1 *
Mean corpuscular hemoglobin concentration (MCHC), g/dL	332.2 ± 6.9	355.9 ± 1.9 *	350.4 ± 1.7 *
Red cell distribution width (RDW), %	11.9 ± 0.5	12.0 ± 0.3	11.6 ± 0.2
Platelets, thousand/μL	1038.5 ± 77.0	1088.7 ± 36.0	1191.9 ± 39.6
Mean platelet volume (MPV), fl	6.14 ± 0.11	6.14 ± 0.07	6.01 ± 0.12
Platelet distribution width (PDW), %	15.7 ± 0.5	16.0 ± 0.1	15.8 ± 0.1
Plateletcrit, %	0.60 ± 0.03	0.64 ± 0.02	0.62 ± 0.02

*—difference with the indicator of the intact group is significant at *p* ˂ 0.05.

## Data Availability

The original contributions presented in this study are included in the article. Further inquiries can be directed to the corresponding author(s).

## Abbreviations

The following abbreviations are used in this manuscript:

BAS	Biologically active substances
CVD	Cardiovascular diseases
RUT	Rutin
MS	Metabolic syndrome
HDL-C	High-density lipoprotein cholesterol
LDL-C	Low-density lipoprotein cholesterol
VLDL-C	Very low-density lipoproteins
AST	Aspartate aminotransferase
ALT	Alanine aminotransferase
TG	Triglycerides
TC	Total cholesterol
AI	Cholesterol atherogenic index
TyG	Triglyceride-glucose index
HOMA-IR	Homeostasis model assessment of insulin resistance
MCV	Mean corpuscular volume
MCH	Mean corpuscular hemoglobin
MCHC	Mean corpuscular hemoglobin concentration
RDW	Red cell distribution width
MPV	Mean platelet volume
PDW	Platelet distribution width
XRD	X-ray diffractometer
NMR	Nuclear magnetic resonance
DMSO	Dimethyl sulfoxide

## Appendix A

**Table A1:** Intensity and position of certain diffraction peaks with relative intensity greater than 5% for the rutin sample.

Pos. [°2Th]	Height [cts]	FWHM Left [°2Th]	d-Spacing [Å]	Rel. Int. [%]
5.213	544.94	0.100	16.94	14.6
7.240	651.53	0.100	12.20	17.6
8.618	394.62	0.100	10.25	8.0
10.466	769.90	0.100	8.45	21.2
14.522	1403.79	0.100	6.09	40.9
14.966	1559.61	0.156	5.91	46.0
15.702	1341.75	0.117	5.64	37.4
16.012	535.02	0.249	5.53	8.0
16.804	2055.48	0.101	5.27	62.5
17.262	495.14	0.150	5.13	6.1
17.842	1042.59	0.169	4.97	25.9
18.570	1053.16	0.112	4.77	26.5
19.565	591.76	0.215	4.53	9.7
19.964	561.73	0.100	4.44	8.4
20.619	919.20	0.183	4.30	21.2
21.712	827.14	0.100	4.09	17.6
22.173	1922.04	0.128	4.01	57.1
22.858	999.96	0.153	3.89	23.9
24.634	510.29	0.356	3.61	5.5
25.492	539.41	0.100	3.49	5.4
25.929	832.59	0.100	3.43	15.5
26.294	3182.90	0.136	3.39	100.0
26.846	2859.81	0.134	3.32	88.0
27.954	621.71	0.100	3.19	6.8
28.360	604.71	0.100	3.14	6.2
29.238	804.52	0.318	3.05	13.0
29.923	746.10	0.100	2.98	10.5
31.238	604.19	0.100	2.86	5.2
32.242	888.04	0.221	2.77	15.6
33.163	783.28	0.187	2.70	12.2
34.464	580.45	0.100	2.60	5.4

**Table A2:** Assignment of signals of the RUT sample in the ^1^H NMR spectrum.

δ, ppm	xH, Multiplicity, J, Hz	Assignment
1.00	3H, d, j = 6.3 Hz	CH3(37)
3.08	2H, m	CH(28), CH(38)
3.26	6H, m	1/2CH2(29), CH(36), CH(39), CH(42), CH(26), CH(27)
3.40	1H, bs	CH(25) + H_2_O
3.72	1H, d, j = 10.6 Hz	1/2CH2(29)
4.35	1H, bs	OH(33)
4.39	2H, m	CH(31), OH(41)
4.53	1H, bs	OH(34)
5.08	1H, d, j = 5.8 Hz	OH(32)
5.12	1H, bs	OH(40)
5.29	1H, d, j = 3.5 Hz	OH(43)
5.35	1H, d, J = 7.4 Hz	CH(23)
6.20	1H, s	CH(2)
6.39	1H, s	CH(6)
6.85	1H, d, j = 8.2 Hz	CH(15)
7.54	1H, d, j = 1.8 Hz	CH(12)
7.55	1H, dd, j = 8.2 Hz, 1.8 Hz	CH(16)
9.18	1H, bs	OH(22)
9.67	1H, bs	OH(21)
10.84	1H, bs	OH(20)
12.60	1H, s	OH(19)

**Table A3:** Attribution of signals for the RUT sample in the NMR spectrum ^13^C{^1^H}.

δ, ppm	xC, Multiplicity, J, Hz	Assignment
17.75	1C, s	CH_3_(37)
67.00	1C, s	CH_2_(29)
68.25	1C, s	CH(36)
70.00	1C, s	CH(27)
70.38	1C, s	CH(28)
70.56	1C, s	CH(25)
71.85	1C, s	CH(38)
74.08	1C, s	CH(26)
75.91	1C, s	CH(39)
76.44	1C, s	CH(42)
93.60	1C, s	CH(6)
98.68	1C, s	CH(2)
100.76	1C, s	CH(31)
101.18	1C, s	CH(23)
103.98	1C, s	C(4)
115.23	1C, s	CH(15)
116.27	1C, s	CH(12)
121.19	1C, s	C(14)
121.60	1C, s	CH(16)
133.30	1C, s	C(9)
144.76	1C, s	C(13)
148.42	1C, s	C(8)
156.43	1C, s	C(11)
156.62	1C, s	C(5)
161.23	1C, s	C(3)
164.08	1C, s	C(1)
177.38	1C, s	C=O(7)
